# Progress on angiogenic and antiangiogenic agents in the tumor microenvironment

**DOI:** 10.3389/fonc.2024.1491099

**Published:** 2024-11-19

**Authors:** Jian Xu, Zhihua Tang

**Affiliations:** Department of Pharmacy, Shaoxing People’s Hospital, Shaoxing, China

**Keywords:** tumor, angiogenic, antiangiogenic agents, anti-angiogenic drugs, antiangiogenic therapy

## Abstract

The development of tumors and their metastasis relies heavily on the process of angiogenesis. When the volume of a tumor expands, the resulting internal hypoxic conditions trigger the body to enhance the production of various angiogenic factors. These include vascular endothelial growth factor (VEGF), fibroblast growth factor (FGF), platelet-derived growth factor (PDGF), and transforming growth factor-α (TGF-α), all of which work together to stimulate the activation of endothelial cells and catalyze angiogenesis. Antiangiogenic therapy (AAT) aims to normalize tumor blood vessels by inhibiting these angiogenic signals. In this review, we will explore the molecular mechanisms of angiogenesis within the tumor microenvironment, discuss traditional antiangiogenic drugs along with their limitations, examine new antiangiogenic drugs and the advantages of combination therapy, and consider future research directions in the field of antiangiogenic drugs. This comprehensive overview aims to provide insights that may aid in the development of more effective anti-tumor treatments.

## 1 Introduction

Tumor angiogenesis is the process through which tumor tissue acquires the nutrients and oxygen necessary for its growth and metastasis by inducing the formation of new blood vessels. In 1971, Folkman was the first to propose a connection between tumor growth and angiogenesis (1, 2). The growth of tumors relies on angiogenesis; in situations of internal hypoxia, tumors have the ability to enhance the expression of several pro-angiogenic factors, thus facilitating the activation of endothelial cells and the process of angiogenesis. This process influences the tumor microenvironment, facilitating tumor growth, invasion, and metastasis (3). Key factors involved in this process include the activation and expression rates of vascular endothelial growth factor (VEGF), hypoxia-inducible factor (HIF), Notch signaling, metabolic reprogramming, and non-coding RNAs (4). Antiangiogenic therapy (AAT) aims to normalize tumor blood vessels by inhibiting these angiogenic signals. Given that these signaling pathways play a critical role in tumor angiogenesis, they have become targets for antiangiogenic therapy (5). At present, AAT has become an important therapeutic approach for a range of illnesses, such as cancer. This paper examines the processes that drive tumor angiogenesis and discusses the progress made in antiangiogenic treatments, with the goal of offering fresh perspectives for addressing tumors and other associated disorders.

## 2 Molecular mechanisms of angiogenesis in the tumor microenvironment

### 2.1 The pro-angiogenic factors

Tumor cells secrete numerous proangiogenic factors that interact with receptors on endothelial cells in blood vessels, promoting their growth, movement, and the development of new vascular structures. Key factors implicated in this process consist of vascular endothelial growth factor (VEGF), fibroblast growth factor (FGF), platelet-derived growth factor (PDGF), and transforming growth factor-α (TGF-α), among others.

#### 2.1.1 Vascular endothelial growth factor (VEGF) family

The VEGF signaling pathway consists of various VEGF ligands and receptors, including VEGF-A, B, C, D, E, F, and placental growth factor, along with receptors such as VEGFR1, 2, and 3. VEGF-A acts as the primary agent that triggers the activation of endothelial cells (ECs) for angiogenesis (6). It attaches to VEGFR2 located on the ECs’ surface, resulting in the phosphorylation of its intracellular domain and the ensuing activation of downstream signaling cascades, including PI3K/AKT and MAPK/ERK. These mechanisms promote the proliferation and migration of ECs, thereby aiding in neovascularization. As a result, VEGF-A has become a significant focus for anti-angiogenic treatments. Additionally, VEGF is an essential factor in angiogenesis that drives the creation of disorganized and primitive blood vessels in various tumor tissues. It initiates various biological processes, many of which are linked to cancer (7); therefore, VEGF has turned into a critical target for anti-angiogenic therapies.

#### 2.1.2 Fibroblast growth factor (FGF) family

The FGFR family encompasses FGFR1 to FGFR5, characterized by a sequence that is notably conserved and homologous. Comprising an extracellular domain, a hydrophobic transmembrane domain, and an intracellular domain responsible for tyrosine kinase activity, FGFR1 to FGFR4 exhibit considerable conservation across these receptors. Conversely, FGFR5 does not possess the intracellular tyrosine kinase domain, leading to its overactivity in the regulatory fibroblast growth factor (FGF) - FGFR signaling pathway (8, 9). The fibroblast growth factor receptor (FGFR) is classified as a receptor tyrosine kinase (RPTK). The activation of the FGFR signaling axis primarily relies on the binding of the ligand FGF to the FGFR dimer, followed by dimerization with fibroblast growth factor receptor substrate 2 (FRS2) and subsequent autophosphorylation. The activation of downstream signaling pathways, including PI3K/AKT and RAS-MAPK, is initiated by this cascade, both of which are crucial for regulating cell proliferation and apoptosis (10, 11).

#### 2.1.3 Platelet-derived growth factor (PDGF)

Platelet-derived growth factor (PDGF) represents an important category of growth factors, predominantly secreted by platelets, and is essential in the mechanisms of cell proliferation, migration, and differentiation (12). This factor typically exists as a homodimer, primarily consisting of PDGF-AA, PDGF-AB, PDGF-BB, PDGF-CC, and PDGF-DD. It exerts its effects through specific receptors, namely PDGFR-α and PDGFR-β. The binding of PDGF to these receptors activates intracellular signaling pathways, including the PI3K/Akt and MAPK pathways, which promote cell proliferation, migration, lumen formation, and the development of new blood vessels (13, 14). PDGF exhibits a dual role in tumor development; it not only supports tumor growth by enhancing the proliferation and migration of tumor-associated fibroblasts but also promotes tumor angiogenesis, thereby increasing the nutrient supply to tumors (15).

#### 2.1.4 Transformation growth factor- α (TGF-α)

Transforming growth factor alpha (TGF-α) is a small, varied factor belonging to the epidermal growth factor (EGF) family. It plays an essential role in controlling cell growth, differentiation, migration, and survival (16). The effects of TGF-α are mediated through its interaction with the epidermal growth factor receptor (EGFR).This binding activates EGFR, resulting in dimerization and autophosphorylation of the receptor, which subsequently triggers downstream signaling pathways (17). Specifically, it activates the Ras/MAPK signaling pathway to promote cell proliferation and survival, as well as the PI3P/Akt pathway to enhance cell survival and inhibit apoptosis (18). Additionally, TGF-α can alter the tumor microenvironment, promoting vasculogenesis and metastasis (19).

### 2.2 Extracellular matrix and angiogenesis

Matrix metalloproteinases (MMPs) represent a group of enzymes that depend on zinc and are responsible for breaking down the extracellular matrix, playing an essential role in the angiogenesis process (20). These enzymes notably aid in tumor progression and metastasis by degrading the matrix that encases the tumor, leading to the release of pro-angiogenic factors such as VEGF, which facilitate the formation of new blood vessels that supply oxygen and nutrients to the tumor (21, 22). Moreover, MMPs promote tumor infiltration by modifying the tumor microenvironment, allowing cancer cells to invade adjacent tissues and access the bloodstream, consequently facilitating tumor invasion and metastasis (23).

### 2.3 Effects of endothelial cells and pericytes

Endothelial cells are specialized cells primarily responsible for forming the inner lining of blood vessels and covering the inner epidermis of both blood and lymphatic vessels. These cells play an essential role in the process of neovascularization. The activation of tumor endothelial cells relies on various signaling pathways. The PI3K/Akt pathway fosters the growth and survival of endothelial cells by regulating their proliferation, survival, and metabolic functions. Additionally, the MAPK/ERK pathway participates in cell proliferation, differentiation, and metastasis, significantly influencing the activation and proliferation of endothelial cells (24). Moreover, the Notch pathway is critical for cell-cell interactions, aiding in the maturation and stability of endothelial cells. Endothelial cells react to angiogenic factors, such as VEGF, which triggers their activation, proliferation, and migration from the surrounding matrix. This sequence of events results in the establishment of new vascular channels through the processes of aggregation, arrangement, and interaction, ultimately leading to the formation of new blood vessels that facilitate tumor growth and dissemination. This process is crucial for tumor progression (25, 26).

Pericytes are specialized cells situated on the lateral aspect of endothelial cells found in the walls of blood vessels, where they play essential roles in vascular development, stability, functionality, and the tumor microenvironment. The relationship between tumor endothelial cells and pericytes is crucial for angiogenesis, tumor progression, and metastasis (27, 28). Growth factors secreted by endothelial cells, such as Vascular Endothelial Growth Factor (VEGF), can stimulate pericyte proliferation and differentiation. Pericytes facilitate endothelial cell differentiation through tight contact with vascular endothelial cells, enabling the formation of a mature vascular lumen. Simultaneously, pericytes contribute to the stabilization and improvement of the structural integrity of blood vessels through the secretion of extracellular matrix elements, which include fibronectin and collagen, as well as multiple growth factors such as Transforming Growth Factor-beta (TGF-β) and Platelet-Derived Growth Factor (PDGF). Activated endothelial cells can increase blood vessel permeability, resulting in fluid and cellular component accumulation within the tumor microenvironment. Nevertheless, pericytes have the ability to control the condition of endothelial cells by means of factors they secrete, aiding in the preservation of standard vascular permeability and thwarting excessive permeability (29). Moreover, the relationship between pericytes and endothelial cells is crucial in the metastatic process of tumor cells. The activation of endothelial cells promotes tumor cell adhesion and enables their traversal across the blood vessel wall by expressing specific cell adhesion molecules. Additionally, pericytes may secrete factors that support tumor cell infiltration during this process. Notably, the deregulation or abnormal activation of pericyte function can lead to alterations in the tumor microenvironment, facilitating tumor cell invasion and metastasis (30).

### 2.4 Signaling pathway

Tumor angiogenesis is a complex biological process affected by various signaling pathways, such as hypoxia induction factor pathway (HIF) (31), vascular endothelial growth factor pathway (VEGF) (32), fibroblast growth factor pathway (FGF) (33), PI3K/Akt pathway (34), Ras/Raf/MEK/ERK pathway (35), Notch signaling pathway (36), transforming growth factor- β pathway (TGF- β) (37), Wnt/β -catenin pathway (38), these pathways are interwoven and synergistic, determine the dynamic balance of tumor angiogenesis. By effectively targeting these key pathways, it may significantly inhibit tumor growth and metastasis, providing new hope for cancer therapy.

### 2.5 Impact on the tumor immune microenvironment

**Figure 1:**
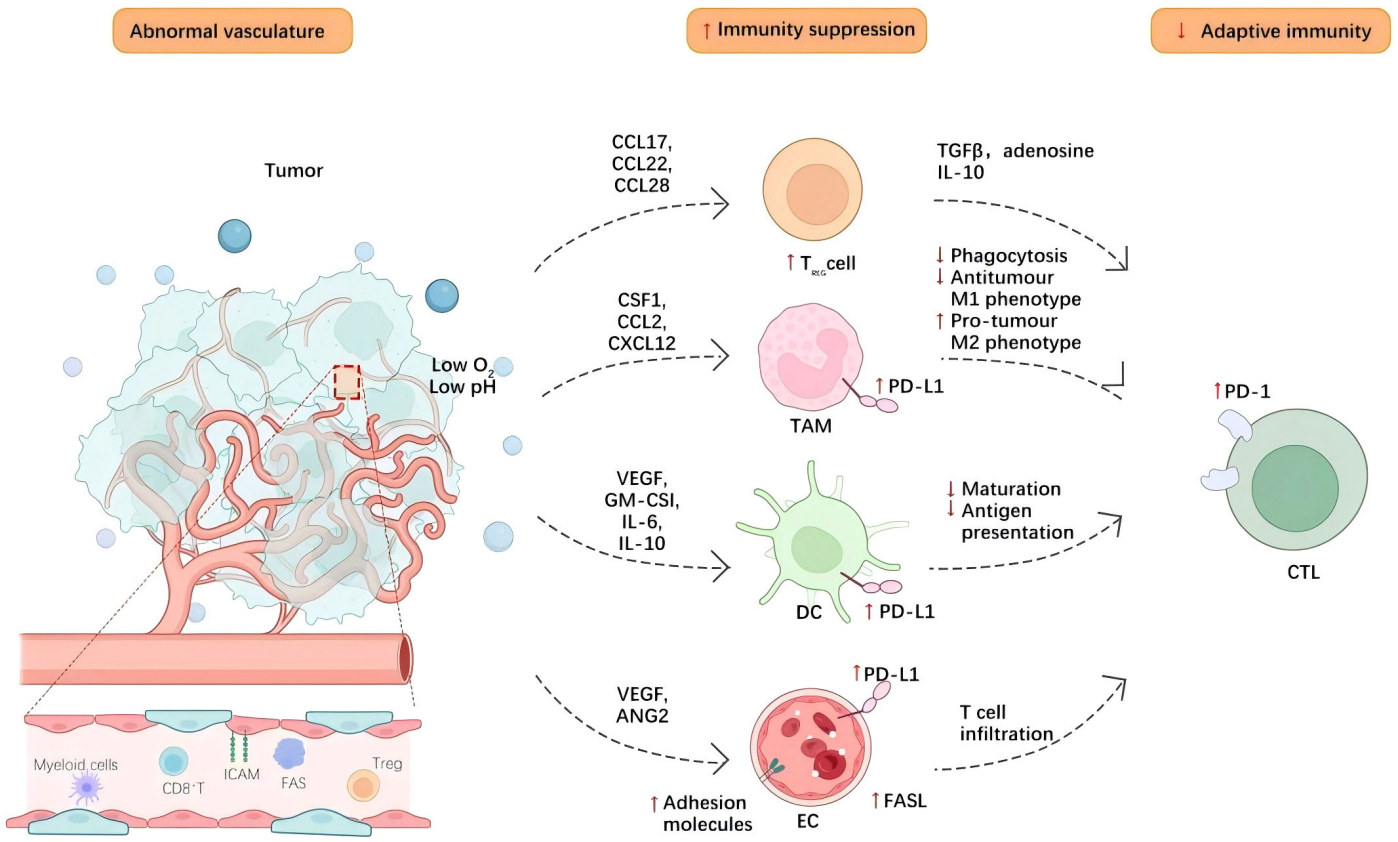
Tumor angiogenesis contributes to an immunosuppressive tumor microenvironment through various mechanisms. Blood vessels within tumors aid in the infiltration of immune cells into the tissue of the tumor, while neovascularization enables lymphocytes, macrophages, and other immune cells to infiltrate the tumor microenvironment more effectively. Cell adhesion molecules, such as selectins and integrins, expressed by tumor endothelial cells, promote interactions between endothelial cells and immune cells, thereby enhancing the adhesion and migration of immune cells. Additionally, neovascularization can induce hypoxia within the tumor microenvironment, leading to functional changes in immune cells. The hypoxic environment can inhibit T-cell function and promote the activation of immunosuppressive cells, including regulatory T cells and tumor-associated macrophages. Concurrently, endothelial cells and tumor cells secrete various cytokines, such as TGF-β-and- IL-10, which can suppress effective antitumor immune responses and further facilitate immune evasion.Hypoxia also elevates the expression of CTLA-4 and TIM-3 on regulatory T cells (Tregs), as well as PD-L1 on myeloid-derived suppressor cells (MDSCs), tumor-associated macrophages (TAMs), and cancerous cells, consequently diminishing the effectiveness of immune-supportive cells. Moreover, endothelial cells within the tumor vasculature, which display diminished amounts of cell adhesion molecules, may trigger endothelial anergy, limiting the ingress of effector T cells into the tumor microenvironment.

The influence of tumor angiogenesis on the tumor immune microenvironment is bidirectional. On one hand, newly formed blood vessels facilitate the infiltration of immune cells; on the other hand, alterations in the tumor microenvironment and abnormal vascular function can impair the functionality of immune cells, thereby promoting tumor growth and metastasis (39) **Figure 1**.

Above, we discuss the molecular mechanisms of angiogenesis in the tumor microenvironment, and we know that they not only provide essential nutrients and oxygen for tumor growth and metastasis, also provide help for tumor cell adaptability and tolerance. To combat this tumor process, the researchers have developed a series of therapeutic strategies for angiogenesis. Next, we will explore the mechanism of action of these antiangiogenic drugs, their clinical application and their potential impact in cancer treatment from the traditional antiangiogenic drugs and the new antiangiogenic drugs.

## 3 Classification and mechanism of action of traditional anti-angiogenic drugs

Traditional anti-angiogenic drugs mainly include macromolecular monoclonal antibodies (mAbs), small molecule tyrosine kinase inhibitors (TKIs), and other categories. Macromolecular monoclonal antibodies are a class of anti-tumor agents that specifically target tumor cells to inhibit their growth or induce cell death. Due to their high specificity and reduced side effects, these antibodies have become a significant class of drugs in cancer treatment. Small-molecule tyrosine kinase inhibitors (TKIs) are targeted therapies employed in the treatment of cancer and other diseases; they block cell proliferation, migration, and survival signals by inhibiting the activity of tyrosine kinases. Additionally, other anti-angiogenic agents include recombinant human vascular endostatin, endothelial cell migration inhibitors, and certain natural traditional Chinese medicines. For further details, please refer to the **Table 1** below.

**Table 1:** Classification and mechanism of action of traditional anti-angiogenic drugs.

Drug name	Clinical identifier	Status	cancer types	mechanism of action	Ref.
Macromolecular mAbs class					
Trastuzumab	NCT06486883 NCT06467357	Phase 2/3	1.HR-positive and HER2- low/Ultralow Advanced Breast Cancer 2,Advanced or Metastatic HER2-expressing Biliary Tract Cancer	Targeted to human epidermal growth factor 2(HER2)	(40, 41)
cetuximab	NCT06412198 NCT06332092	Phase 1/2	1.Metastatic Colorectal Cancer Harboring KRAS G12C Mutations 2.Neck Squamous Cell Carcinoma	Targeted to the growth factor receptor mAb(GFR)	(42, 43)
bevacizumab	ChiCTR2400087075 NCT06509906 ChiCTR2400086343	Phase 2/3	1.Advanced non-small cell lung cancer 2.Advanced Solid Tumor 3.Hepatocellular carcinoma	Targeted tovascular endothelial growth factor(VEGF)	(44–46)
Pembrolizumab	ISRCTN73427832 NCT06502080 NCT06498518	Phase 2	1.Lung cancer/advanced squamous cell lung cancer 2.Bilary Tract Cancer 3.Metastatic Pancreatic Cancer	Programmed cell death protein 1(PD-1)	(47–49)
rituximab	NCT06510309 NCT06508658	Phase 2/3	1.Marginal Zone Lymphoma 2.Diffuse Large B-Cell Lymphoma	CD20	(50, 51)
Small-molecule tyrosine kinase inhibitors					
Erlotinib	ChiCTR2400085138 KCT0009473 CTRI/2024/04/065574	Phase 2/3/4	1.Advanced non-small-cell lung cancer 2.Metastatic colorectal cancer 3.oral cancer	Epidermal growth factor receptor (EGFR) inhibitor	(52–54)
Gefitinib	CTRI/2024/01/061126 ChiCTR2400085138	Phase 4	1.Lung Cancer 2.Advanced non-small-cell lung cancer	Epidermal growth factor receptor (EGFR) inhibitor	(55, 56)
Osimertinib	NCT06498986 NCT06477055	Phase 2	1.Advanced or metastatic non-small-cell lung cancer 2.nonsmall-cell lung cancer	Epidermal growth factor receptor (EGFR) inhibitor	(57, 58)
Sorafenib	NCT06474663 CTIS2024-512212-21-00	Phase 2/3	1.Relapsed and refractory acute leukemia 2.Advanced liver cancer	Tyrosine kinase receptor (TKR) inhibitor	(59, 60)
Sunitinib	CTIS2022-503105-38-00	Phase 3	Renal cell carcinoma of advanced or metastatic traits	Tyrosine kinase receptor (TKR) inhibitor	(61)
Imatinib	CTIS2023-508463-71-00 CTIS2022-503068-34-00	Phase 2/3	1.acute lymphoblastic leukemia 2.Advanced gastrointestinal stromal tumor	The BCR-ABL inhibitor	(62, 63)
Dasatinib	NCT06390319 NCT06355037 CTIS2023-507537-16-00	Phase 1/2	1.acute lymphoblastic leukemia 2.mammary cancer 3.multiple myeloma	The BCR-ABL inhibitor	(64–66)
Nilotinib	CTIS2023-510434-83-00 CTIS2024-510947-71-00	Phase 3/4	1.chronic myeloid leukemia 2.chronic myeloid leukemia	The BCR-ABL inhibitor	(67, 68)
Regorafenib	ChiCTR2400086011	Phase 1	1.Colorectal Cancer Liver Metastases	Vascular endothelial growth factor receptor (VE GF R) inhibitors	(69)
Other types					
Endostar	ChiCTR2400085777 NCT06301828	Phase 2/4	1.nonsmall-cell lung cancer 2.Advanced gastrointestinal tract tumors	Recombinant human vascular endostatin	(70, 71)
Rapamycin	ChiCTR2300070701	Phase 3	Peripheral neuroblastoma tumors	endothelial cell migration inhibitors	(72)
Mitomycin	KCT0009491 CTRI/2024/04/066329	Phase 2 /Post Marketing Surveillance上市后监督	1.non-muscle-invasive urinary bladder cancer 2.Conjunctival surface tumor in the human eye surface tumor of the conjunctiva of the human eye	endothelial cell migration inhibitors	(73, 74)
Paclitaxel	NCT06519591 NCT06513455 ChiCTR2400086859	Phase 1/2	1.Gastric Cancer With Peritoneal MetastasisPhase 2.cancer of pancreas 3.esophageal squamous cancer	Other natural plants	(75–77)
quercetin	NCT06355037 IRCT20230509058138N1 NCT05724329	Phase 2/3	1.mammary cancer 2.mammary cancer 3.Head and Neck Squamous Cell Carcinoma	Other natural plants	(78–80)

## 4 New progress in the research of anti-angiogenic drugs

In the treatment of solid tumors, conventional anti-angiogenic therapies that target growth factors, primarily anti-VEGF therapies, have not met expectations regarding efficacy and patient survival. This shortfall can be attributed to both intrinsic and acquired treatment resistance, as well as complications arising from adverse drug reactions. Consequently, current research on anti-angiogenic drugs has focused on strategies to mitigate or delay drug resistance while simultaneously enhancing drug efficacy and reducing or eliminating toxic side effects.

### 4.1 Drug development based on new targets

Drug development based on new targets is one of the important directions of cancer therapy research in recent years. Researchers continue to explore new angiogenesis-related targets and molecular mechanisms.

#### 4.1.1 gC1qR

The gC1qR protein, referred to as P32/C1qBP/HABP1, is a versatile protein that is found at elevated levels in various types of cancer and possesses prognostic significance. It governs multiple related signaling pathways, such as the Wnt/β-catenin, PKC-NF-κB, and Akt/PKB pathways, which may contribute to the promotion of apoptosis in tumor cells and possess antiangiogenic properties (81). In studies conducted by Peerschke E (82), both *in vitro* and *in vivo* experiments demonstrated that the specific monoclonal antibody gC1qR mAb 60.11 could inhibit tumor growth in mouse models of mesothelioma, consequently reducing the proliferation and spread of cancer cells, thus positioning it as a novel target for treating mesothelioma. Currently, several preclinical studies exploring gC1qR-targeted interventions, which include approaches like monoclonal antibodies, chimeric antigen receptor T cell (CAR-T) therapy, and tumor vaccination, are being systematically pursued, yielding promising preclinical therapeutic outcomes (83). The investigation into the targeting of gC1qR marks an emerging area in cancer immunotherapy, offering both prospects and challenges for the progression of clinical treatments.

#### 4.1.2 PDGF

Platelet-derived growth factor (PDGF) represents a class of inhibitors that target PDGF and its receptors (PDGFR). PDGF binds to its receptor (PDGFR), initiating the cell signal transduction pathway and promoting angiogenesis and tumor cell proliferation (84). For instance, Imatinib was initially utilized as a therapeutic agent for acute myeloid leukemia and chronic myeloid leukemia, with its mechanism of action involving the inhibition of PDGFR, thereby demonstrating antitumor activity against certain solid tumors. Moreover, multitargeted tyrosine kinase inhibitors like Sunitinib and Sorafenib not only inhibit the vascular endothelial growth factor receptor (VEGFR) but also target PDGFR, demonstrating their efficacy in treating renal cell carcinoma (85) and hepatocellular carcinoma (86). Novel small-molecule targeted drugs have also entered preclinical or clinical trials, aiming to more precisely inhibit the PDGF/PDGFR signaling pathway without affecting other critical growth factor signaling pathways, thus attracting the attention of developers. Despite some advancements in the development of antitumor drugs targeting PDGF, challenges persist. For example, certain tumors have developed resistance to PDGF intervention (87, 88). Moreover, the specific roles and mechanisms of PDGF in various cancers, beyond lung and kidney cancers, require further elucidation.

#### 4.1.3 MMPs

Metalloproteinases (MMPs) are essential in the processes of angiogenesis and the spread of tumors. In the past few years, there have been notable improvements in the study of inhibitors for metalloproteases (89). Early MMP inhibitors, such as Marimastat, demonstrated some efficacy in preclinical and certain clinical trials; however, their widespread use has been limited due to side effects and efficacy concerns (90). The new generation of small-molecule MMP inhibitors aims to optimize selectivity and pharmacokinetic properties, with examples including Siramesin, BMS-981016, and BMS-845145. These novel compounds exhibit enhanced drug resistance and anti-tumor activity across various cancer types (91). Additionally, inhibitors targeting specific MMPs are under development, such as selective inhibitors against MMP-9, which may offer greater effectiveness and reduced side effects (92). While the prospects for new MMP targets are promising, challenges such as drug resistance and side effects persist. Consequently, enhancing the selectivity and relevance of these inhibitors remains a primary focus of current research and development.

#### 4.1.4 miRNA

Certain microRNAs (miRNAs) can regulate the expression of angiogenesis-related genes by binding to target mRNA, and current drugs targeting miRNAs are under development. miRNA replacement therapy involves introducing a tumor-suppressor miRNA that has been deleted or impaired, such as targeting the deleted miRNAs like miR-34a and miR-29. This area of research is actively progressing (93). MRX 34 is a nanoparticle medication derived from human miR-34a, specifically formulated for the treatment of liver cancer and is presently undergoing assessment for its safety and effectiveness in clinical trials (94). TargomiRs represent an endogenous miRNA repair therapy that utilizes liposome delivery systems encapsulating miRNA combined with targeting mechanisms (such as antibodies) to specifically target tumor cells and enhance the expression of tumor-suppressor miRNAs (95). AntagomiRs, developed in the form of antisense RNA, function by inhibiting specific tumor-promoting miRNAs (e.g., miR-21) (96). Ongoing studies on miR-155 inhibitors are also anticipated to facilitate treatment for lymphoma and some sarcomas (97). Novel tumor drugs based on miRNAs offer new hope for cancer treatment (93, 98, 99). However, despite their considerable potential, miRNA drugs face several challenges. For instance, the effective delivery of miRNAs or their inhibitors is a critical issue due to the biocompatibility and tissue specificity of the drugs themselves. Additionally, the specificity and durability of these drugs require attention to ensure the development of selective miRNA therapies that avoid potential adverse effects on normal cells, while also ensuring the stability and longevity of the drug within the body to prevent early degradation (100).

### 4.2 Multi-target inhibitors

Tumors are complex diseases that often involve the metabolic processes of multiple genes and signaling pathways. Traditional single-target drugs exhibit limited efficacy regarding tumor specificity and the development of drug resistance. In contrast, this new class of multi-target tumor inhibitors can simultaneously target multiple signaling pathways or molecules, thereby enhancing drug efficacy and mitigating or delaying the onset of drug resistance.

Multi-target Tyrosine Kinase Inhibitors (MTKIs) are a type of medication that can inhibit several tyrosine kinases at once. These compounds affect the growth, proliferation, angiogenesis, and metastasis of tumor cells, thus having a broad impact on the tumor microenvironment and playing a vital part in cancer therapy. For instance, Cabozantinib can inhibit the kinase activity of VEGFR, MET, and other receptors, and is utilized in the treatment of thyroid adenocarcinoma (101) and renal cell carcinoma (102). Similarly, Lapatinib targets EGFR and HER2, primarily serving patients with HER2-positive breast cancer (103, 104). Sorafenib inhibits RAF kinase, VEGFR, and PDGFR concurrently, and is predominantly used for hepatocellular carcinoma and renal cell carcinoma (105, 106). Lenvatinib, which targets VEGFR, FGFR, and PDGFR, is indicated for thyroid cancer and hepatocellular carcinoma (60, 107). Apatinib focuses on the inhibition of VEGFR-2 and other related kinases, primarily treating advanced gastric cancer and liver cancer (108, 109). Ponatinib, which targets BCR-ABL and various other kinases, demonstrates significant efficacy in certain leukemia treatments (110). Entrectinib serves as an oral inhibitor targeting the tyrosine kinases TRKA/B/C, ROS1, and ALK, and is utilized in the treatment of solid tumors that harbor NTRK gene fusions as well as non-small cell lung cancer featuring ROS1 mutations (111). European Organisation for Research and Trearch and Treatment of Cancer phase II trial 1317 The ‘CaboGIST’ clinical trial showed that (112), cabbotinib (a multitarget TKI inhibitor) achieved an 82% disease control rate in the treatment of GISTs, which confirmed preclinical findings. The clinical application of anlotinib (a novel multi-target TKI inhibitor) by SunY (113) in patients with advanced solid tumors showed that Anlotinib shows controlled toxicity, long circulation and broad-spectrum antitumor potential.

### 4.3 Novel antibody drugs

In recent years, with the rapid development of biomedical technology, some new therapeutic strategies have gradually emerged, including Antibody-drug conjugates (ADCs), Peptide-drug conjugates (PDCs), Bispecific antibodies (BsAb), etc., which provide new hope for the treatment of cancer and other diseases.

#### 4.3.1 Antibody-drug conjugate

Antibody-drug conjugates (ADCs) combine antibodies with cytotoxic drugs to deliver toxins directly to the tumor microenvironment through targeted mechanisms, thereby reducing side effects (114, 115). The formation of new carrier proteins and the targeting of drugs to the tumor microenvironment can effectively enhance tumor distribution or activation, thus improving the anti-cancer activity against difficult-to-treat tumors (116). ADC molecules combine the accuracy of antibody-targeted tumor antigen recognition with powerful cytotoxic components, leading to targeted delivery systems for cancers. A key benefit of ADCs is their capacity to enhance the therapeutic index by precisely directing the cytotoxic payload to tumor tissues, which in turn lowers the minimum effective dose (MED) and notably reduces the incidence of adverse events (117). See **Table 2** for details.

**Table 2:** Antibody-drug conjugates.

Study drug	Antibody	drug	Cancer types	Status	Clinical trial identifier	Ref.
SG	Sacituzumab	govitecan	Advanced epithelial carcinoma	phase 1/2	NCT01631552	(118)
DS-8201	Trastuzumab	deruxtecan	advanced breast and gastric or gastro-oesophageal tumours	phase 1	NCT02564900	(119)
Dato-DXd	datopotamab	deruxtecan	advanced non-small-cell lung cancer	phase 1	NCT03401385	(120)
CLDN6-23-ADC	CLDN6-23-mAb	monomethyl auristatin E	CLDN6-Positive Solid Tumors	preclinical	--	(121)
T-DXd	Trastuzumab	deruxtecan	HER2-Expressing Solid Tumors	phase 2	NCT04482309	(41)
BL-B01D1	SI-B001	ED04	locally advanced or metastatic solid tumours	phase 1	NCT05194982	(122)
MRG003	humanized immunoglobulin G1 monoclonal antibody	monomethyl auristatin E	Advanced Solid Tumors	phase 1	NCT04868344	(123)
SAR408701	tusamitamab	ravtansine	Advanced Solid Tumors	phase 1	NCT02187848	(124)
HER3-DXd	Patritumab	deruxtecan	HER3-expressing advanced breast cancer	phase 1/2	NCT02980341	(125)
DNIB0600A	lifastuzumab	vedotin	platinum-resistant ovarian cancer	phase 2	NCT01991210	(126)
SG	sacituzumab	govitecan	Breast Cancer	phase 1	NCT04039230	(127)
SG	Sacituzumab	govitecan	Metastatic Triple Negative Breast Cancer	phase 3	NCT02574455	(128)
BMS-986148	nivolumab	mesothelin-directed targeting of the cytotoxic	Advanced Solid Tumors	phase 1/2	NCT02341625	(129)
MORAb-202	Farletuzumab	Eribulin Mesylate	Advanced Solid Tumors	phase 1	NCT03386942	(130)
RC48-ADC	Trastuzumab	monomethyl auristatin E	Locally Advanced or Metastatic Urothelial Carcinoma	phase 2	NCT03507166	(131)

#### 4.3.2 Peptide-drug conjugates

Peptide-drug conjugates (PDCs) represent an emerging class of targeted therapies composed of a carrier peptide, a toxic payload, and an adaptor that links the payload to the peptide. These conjugates demonstrate enhanced tumor permeability and increased specificity for targeted cancers (132). The first PDC approved by the FDA is Lu-177 DOTA-TATE (Lutatera), which is a conjugate of a radionuclide and the peptide octreotide. Singh S (133) and other studies have shown that the first-line treatment of 177Lu-Dotatate in combination with octreotide LAR significantly prolongs the median progression-free survival of patients with grade 2 or grade 3 advanced gastroenteropancreatic neuroendocrine tumors.For instance, Melphalan flufenamide(a first-in-class alkylating peptide-drug conjugate), In an open-label, phase III LIGHTHOUSE studyp (134)Shows, it has a good efficacy and a high safety profile in the treatment of triple-class refractory relapsed/refractory multiple myeloma (RRMM).ANG1005 is a brain-penetrating peptide-drug conjugate that consists of three paclitaxel molecules covalently linked to Angiopep-2. Research by Kumthekar P (135) and others indicates that ANG1005 exhibits high activity in breast cancer patients with leptomeningeal carcinoma and recurrent brain metastases.

#### 4.3.3 Bispecific antibody

Bispecific antibodies (BISpecific Antibodies) are a class of antibodies capable of simultaneously binding to two distinct target antigens. These antibodies can recognize and attach to two different molecules, typically targeting tumor surface antigens and the surfaces of immune cells. See **Table 3** for details.

**Table 3:** Bispecific antibody.

Study drug	target	Cancer types	Status	Clinical trial identifier	Ref.
Epcoritamab	CD3×CD20 T-cell	Relapsed or Refractory Large B-Cell Lymphoma	phase 1/2	NCT03625037	(136)
Mosunetuzumab	CD20×CD3 T-cell	relapsed or refractory follicular lymphoma	Phase 2	NCT02500407	(137)
Talquetamab	CD3×GPRC5D T cell	Multiple Myeloma	Phase 1	NCT03399799	(138)
Glofitamab	CD20 on B cells and CD3 on T cells	Relapsed or Refractory B-Cell Lymphoma	Phase 1	NCT03075696	(139)
Teclistamab	BCMA and CD3 on T cells	relapsed or refractory multiple myeloma	Phase 1	NCT03145181	(140)
JNJ-64407564	GPRC5DxCD3 T-cell	multiple myeloma	Phase 1	NCT03399799	(141)
GEN1046	PD-L1 and 4-1BB	Advanced Refractory Solid Tumors	Phase 1	NCT03917381	(142)
Odronextamab	CD20 on B cells and CD3 on T cells	CD20-positive B-cell malignancies	Phase 1	NCT02290951	(143)
cadonilimab	PD-1 and CTLA-4	advanced solid tumours	Phase 1	NCT03852251	(144)
Zanidatamab	two non-overlapping domains of HER2	locally advanced or metastatic HER2-expressing or HER2-amplified cancers	Phase 1	NCT02892123	(145)
ivonescimab	PD-1 and VEGF	advanced immunotherapy-naive NSCLC	Phase 1	NCT04900363	(146)

#### 4.3.4 Other novel targeted antibodies

Other novel targeting antibodies, such as T-cell engager antibodies (TEGEV), are designed to direct T cells to tumors, thereby enhancing the immune system’s attack on cancer. Bispecific T-cell adapter (BiTE) therapy has emerged as one of the most promising treatments (147). For instance, Blinatumomab, an exemplary medication in BiTE therapy, has shown considerable effectiveness in treating advanced acute lymphoblastic leukemia (148). Furthermore, Tarlatamab (AMG 757), a molecule that conjugates dual-specific T cells and interacts with DLL3 and CD3, enhances tumor lysis mediated by T cells and has demonstrated promising outcomes in treating small cell lung cancer (149). Despite the advancements from traditional monoclonal antibodies to the next generation of antibody drugs, several challenges remain. First, drug resistance poses a significant obstacle; thus, developing therapies that address resistance mechanisms is a critical direction for future research. Second, the side effects of antibody drugs, particularly immune-related adverse reactions, require careful attention. The development of more selective antibodies aimed at minimizing these adverse reactions is also a key area of ongoing research.

Emerging anti-tumor treatment strategies such as Antibody-drug conjugates (ADCs), Peptide-drug conjugates (PDCs) and Bispecific antibodies (BsAb) not only have their own characteristics in mechanism, also show good therapeutic effects in clinical application, and gradually show their unique advantages and broad application prospects. However, it is worth noting that monotherapy is often difficult to meet the treatment needs of complex tumor microenvironment and specific tumors, and therefore, the combined application of drugs has gradually become an important research direction. Next, we will explore the strategies for combining antiangiogenic agents with other drugs and their potential advantages in cancer treatment.

### 4.4 Combination with antiangiogenic agents

#### 4.4.1 Combination of anti-VEGF antibodies with other antitumor agents

Studies have demonstrated that the combination of Bevacizumab with chemotherapy agents, such as cisplatin and paclitaxel, can enhance therapeutic efficacy, particularly in non-small cell lung cancer, colorectal cancer, breast cancer, and other malignancies (150–154). The anti-VEGF antibody has been shown to increase tumor sensitivity to radiotherapy, and its combination with radiotherapy has yielded improved outcomes in liver cancer and brain tumors. Wang et al. (155) tracked 100 patients with esophageal cancer and found that the combination of Bevacizumab with gemcitabine and cisplatin resulted in significant clinical efficacy, effectively improving patient survival rates. Wilke et al. (156) conducted a randomized, placebo-controlled, double-blind phase 3 trial across North America, South America, and 170 countries in Europe, Asia, and Australia. After two years of clinical follow-up, they observed that the combination of paclitaxel and Bevacizumab for advanced gastric cancer outperformed the placebo and paclitaxel group. Additionally, Pfisterer et al. (157) explored the use of Bevacizumab in conjunction with platinum-based agents for recurrent ovarian cancer and established that the combination of carboplatin-PEGylated liposomal doxorubicin and Bevacizumab signifies a novel standard treatment choice for patients with platinum-eligible recurrent ovarian cancer. Eskander and colleagues (158) showed in a randomized phase 3 trial that was both double-blind and placebo-controlled that incorporating Pabolizumab into the standard chemotherapy regimen notably extended progression-free survival among patients suffering from advanced or recurrent endometrial cancer, in comparison to chemotherapy without the addition of Pabolizumab. Furthermore, after five years of clinical observation, de Castro Jr. et al. (159) discovered that patients with locally advanced or metastatic non-small cell lung cancer experienced enhanced treatment outcomes when treated with a combination of Pabolizumab and chemotherapy.

#### 4.4.2 The combination of small-molecule tyrosine kinase inhibitors (TKIs)

Tyrosine kinase inhibitors (TKIs), such as Sorafenib and Sunitinib, when combined with vascular endothelial growth factor (VEGF) inhibitors, can target multiple receptors and inhibit the proliferation and angiogenesis of tumor cells, resulting in a synergistic effect. In a single-arm phase II study, McNamara et al. (160) revealed that Axitinib showed promising and tolerable clinical efficacy in individuals with hepatocellular carcinoma (HCC) who had previously received VEGF inhibitors during the advanced stages of their illness. In the phase 3 COSMIC-312 study, Cabozantinib combined with Atezolizumab significantly improved progression-free survival compared to Sorafenib, showing enhanced disease control and reduced primary progression (161). Motzer and colleagues (162) discovered that the combination of Avelumab (a PD-L1 inhibitor) with Axitinib significantly extended progression-free survival and resulted in an objective response as the first-line treatment for advanced renal cell carcinoma. In an open-label phase 3 trial (KEYNOTE-426), Pembrolizumab combined with Axitinib significantly extended overall survival and progression-free survival, while also increasing objective response rates in previously untreated patients with advanced renal cell carcinoma (163). Long-term follow-up data revealed that the combination of Navolimab and Cabozantinib significantly enhanced overall survival in individuals suffering from advanced renal cell carcinoma, showcasing notable targeting and antitumor effects, with a median overall survival of 32.9 months (164). Xia et al. (165) concluded from a phase II clinical trial that perioperative Carilizumab combined with Apatinib showed promising efficacy and manageable toxicity in patients with resectable liver cancer. The phase 3 trial (CARES-310) conducted by Qin et al. (166) across 95 centers in 13 countries confirmed that Carilizumab provided statistically and clinically significant benefits in progression-free survival and overall survival, establishing it as a new effective first-line treatment option for this patient population. A phase Ib/IIa study showed that (167), allotinib (a multi-target TKI enzyme inhibitor) combined with natuluzumab (immune checkpoint inhibitor) showed a controlled safety and promising efficacy signal in the clinical treatment of non-small cell lung cancer (NSCLC). A phase Ib study (168) showed that vorolanib (a multi-target TKI inhibitor) and pembrolizumab or nivolumab (immune checkpoint inhibitor) showed lower toxicity and promising efficacy in the clinical treatment of advanced solid tumors.

#### 4.4.3 Combination of multiple-target inhibitors

Multi-target anti-angiogenic drugs, such as Cabozantinib, target VEGFR, MET, and other pathways, and their combination with immunotherapy, such as PD-1 antibodies, can significantly enhance anti-tumor efficacy. The preliminary interim evaluation of the randomized phase 3 KEYNOTE-811 study indicated that pabolizumab was evaluated in conjunction with trastuzumab, along with fluoropyrimidine and platinum-based chemotherapy, for individuals with HER2-positive metastatic gastric and gastroesophageal junction cancer, as opposed to a placebo. This interim analysis further demonstrated that the first-line combination of pabolizumab with trastuzumab and chemotherapy was superior to placebo in terms of progression-free survival (169). Margetuximab, an innovative Fc-engineered monoclonal antibody targeting HER2, boosts antibody-dependent cytotoxicity and may promote interaction between innate and adaptive immune systems. This effect is further intensified through checkpoint inhibition, leading to enhanced antitumor responses. Catenacci et al. first reported the combination of anti-HER2 and anti-PD-1 therapies in treating gastroesophageal adenocarcinoma, confirming the safety and efficacy of this approach through phase 3 of KEYNOTE-811 and phase 2 studies of INTEGA (170). Additionally, Shah et al. (171) utilized penpulimab and anlotinib in combination with albumin-bound paclitaxel/gemcitabine (PAAG) in patients with first-line metastatic pancreatic cancer, demonstrating the promising clinical efficacy of anti-angiogenic therapy combined with chemotherapy in this patient population. Conforti et al. (172) discussed the effectiveness and safety of using avelumab, an anti-PD-L1 inhibitor, in conjunction with the anti-angiogenic drug acacitinib for individuals suffering from advanced B3 thymoma and thymic carcinoma.

#### 4.4.4 Combination of nanotech drug delivery systems

The combination of anti-angiogenic drugs and chemotherapy agents utilizing nanotechnology (such as liposomes and polymer nanoparticles) can enhance drug bioavailability and targeting, reduce side effects, and improve anti-tumor efficacy (173). See **Table 4** for details.

**Table 4:** combination of Nanotech drug delivery systems.

Drug name	Payload	Nanovector types	Particle size	Surface property	Drug loading	Encapsulation efficiency	Targeted drug	Drug release	Efficacy evaluation	Refs.
LyP-1-LMWH-Qu	Quercetin and low-molecular-weight heparin	Self-assembled polymer nanoparticles	172.2 ± 2.8 nm	amphipathy	13.0%	53.5%	LyP-1 peptide	_	Tumor growth was reduced by 72.5%	(174)
Sora@PEDF-NPs	Sorafenib and PEDF	Polymer-based nanoparticles	289.1 ± 2.11 nm	Amhiphilic, negative charge	13.19 ± 0.20%	76.1 ± 3.52%	PEG-PLGA	48h(80%)	Tumor suppression was close to 70%	(175)
LD-SDN	sorafenib (SRF) and Dihydroartemisinin (DHA)	lipid nanoparticles	126.5±1.33 nm	negative charge	13.5±0.85%	94.5±1.62%	ApopB-10	24h(~37%) 60h(~75%)	4-fold decrease in tumor volume than those of control	(176)
AFT-PLN@MAp	Afatinib and NIR PLN	persistent luminescence nanoparticles	225nm	almost electrically neutral	15%	_	MAGE-A3 (MAp)	12h(almost)	Decrease tumor size of almost fourfold	(177)
OSI + SEL NP	osimertinib and selumetinib	Nanoparticles for co-delivery	43 nm	negative charge	13%	80%	PEG-S-SEL	25h(~60%) 50h(~8080%)	Decrease tumor size and weight of almost fourfold	(178)
cRGDyk-anlotinib-RM	Anlotinib and RM	reduction-sensitive nanomicelle	30 nm	negative charge	8.98%	98.64%	cRGDyk	24h(50%) 72h(80%)	a 2-fold decrease in tumor volume than those of control	(179)
PEG/PTX-OSI-PLGA9000 EENP	Osimertinib and paclitaxel	erythrocyte-shaped electrosprayed nanoparticles	846.9±65.72nm	positive charge	3.75%	>80%	PEG	24h(80%)	Decrease tumor size of almost 90%	(180)

#### 4.4.5 Antiangiogenic drugs were used in combination with cell therapy

**Figure 2:**
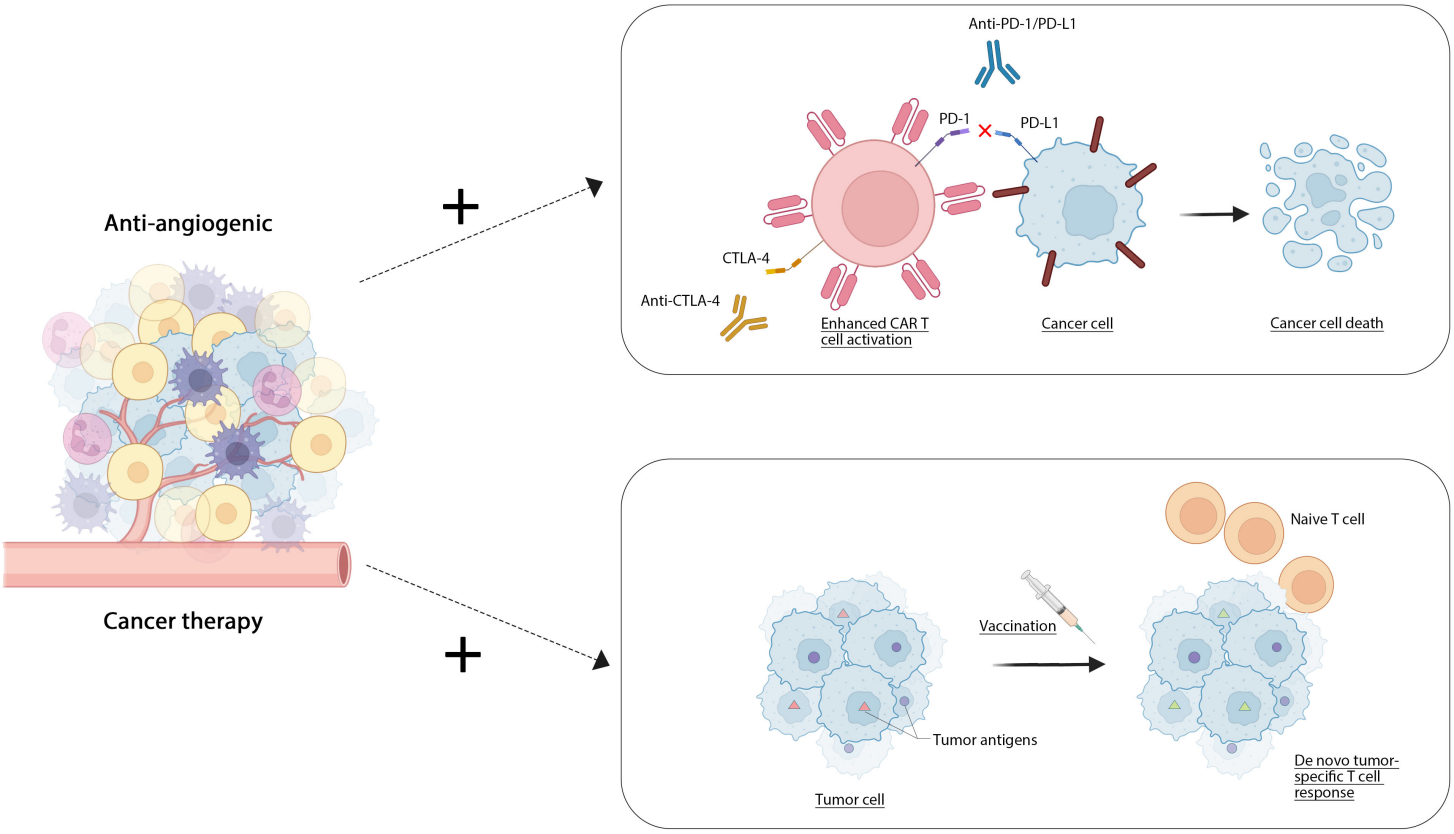
Antiangiogenic drugs combined with cell therapy.

Cell therapy, a form of immunotherapy, utilizes living cells as therapeutic agents to repair, replace, or enhance cellular function, thereby exerting a therapeutic effect. This category includes CAR-T cell therapy, T-cell immunotherapy, and cancer vaccines (181). CAR-T cell therapy involves the modification of a patient’s own T cells through genetic engineering to specifically identify and attack tumor cells. FDA-approved products such as Kymriah and Yescarta have demonstrated significant efficacy in treating acute lymphoblastic leukemia and large B-cell lymphoma, respectively (182). T-cell immunotherapy (183) enhances T cells by introducing them *in vitro* to the patient, employing technologies such as CRISPR/Cas9 (184) for optimization. Cancer vaccines are formulated using tumor cells or tumor-associated antigens, aimed at activating the body’s immune system to combat tumors. The combination of traditional anti-angiogenic drugs with emerging anti-tumor therapies can produce a synergistic effect, thereby enhancing overall efficacy. Combining anti-angiogenic drugs and cell therapy can improve the therapeutic effect on the one hand. The first use of anti-angiogenic drugs can improve the infiltration of immune cells (such as T cells, NK cells, etc.) in the tumor, and improve the effect of cell therapy. At the same time, antiangiogenic drugs can change the tumor microenvironment, reduce the immune escape ability of tumor cells, and promote the activity of T cells in cell therapy. On the other hand, by reducing drug resistance. The combination of these two treatments can inhibit adaptive changes in tumor cells, slow down drug resistance, and improve efficacy. Finally, the combination may allow lowering the dose required for cell therapy but maintain efficacy (185). At present, several clinical trials are evaluating the effect of combining antiangiogenic drugs with cell therapy, mainly in solid tumors such as mamomas, lung cancer, as well as some hematologic malignancies (such as leukemia, lymphoma), and have achieved good results. Zhang J and colleagues (186) engineered gene-specific targeted CAR-T cells using non-viral approaches via CRISPR-Cas9 technology. Their research indicated that employing non-viral anti-CD19 CAR-T cells integrated with PD1 resulted in an improved safety profile for patients suffering from relapsed or refractory invasive B-cell non-Hodgkin lymphoma (r/rB-NHL), characterized by a reduced rate of mild cytokine release syndrome (CRS) and the absence of neurotoxic effects. Elpamotide functions as an epitope peptide associated with the vascular endothelial growth factor receptor 2 (VEGFR-2), stimulating cytotoxic T lymphocytes (CTL) to target and eliminate VEGFR2-expressing endothelial cells. A single-arm phase II trial showed that gemcitabine with an antiangiogenic vaccine had good resistance and moderate antitumor effects in patients with advanced or recurrent biliary tract cancer (187).Wang and colleagues developed a ferritin nanoparticle platform modified with SpyCatcher that facilitates straightforward and stable covalent attachment to tumor-specific antigens containing SpyTag. They validated through experiments that ferritin nanoparticles loaded with either the HPV16 oncogene E7 peptide antigen or neoantigens derived from MC38 tumor mutations provoke a more robust antigen-specific cytotoxic T lymphocyte (CTL) response and significantly reduce the growth of tumors associated with E7 or MC38. Additionally, the therapeutic impact was further amplified when paired with a PD-1 checkpoint inhibitor (188) **Figure 2**.

Antiangiogenic agents have been utilized in conjunction with oncolytic cells to enhance the anti-tumor effect (189, 190). These agents reduce tumor blood flow, thereby improving the infection efficiency of the oncolytic virus (191). A lower blood flow rate allows the oncolytic virus to remain within the tumor for a longer duration, facilitating its function. Additionally, antiangiogenic agents may decrease the immunosuppressive environment within the tumor, promoting more efficient replication of the oncolytic virus and its spread to adjacent tumor cells (192). Furthermore, these agents can inhibit tumor recurrence. To some extent, antiangiogenic drugs reduce immunosuppressive factors in the tumor microenvironment, thereby supporting the immune response triggered by oncolytic viruses and aiding in the prevention of tumor recurrence and metastasis (193). Concurrently, the combination of antiangiogenic drugs with oncolytic cells can enhance the immune response (194). The application of oncolytic cells induces the release and presentation of tumor antigens. In this context, antiangiogenic drugs can further opsonize the tumor microenvironment, thereby promoting the immune system’s response and anticancer activity (195). In recent years, several clinical trials have assessed the combination of anti-angiogenic drugs and oncolytic viruses, demonstrating promising efficacy in various refractory solid tumors, including pancreatic and liver cancers (196, 197). Endostatin, an angiogenesis inhibitor, plays a crucial role in tumor growth, tissue invasion, and metastasis. Some researchers have employed adenoviruses containing the human endostatin gene for intratumoral injection without any reported safety concerns. During treatment, serum levels of endostatin increased, and patients exhibited good tolerance (198). Oncolytic virus (OV) therapy represents a novel approach to cancer treatment. Chesney et al. conducted the first randomized trial evaluating the combination of T-VEC and ipilimumab for melanoma, which assessed the addition of oncolytic viruses to checkpoint inhibitors. The results indicated that the combination of Talimogene Laherparepvec and ipilimumab exhibited significantly higher efficacy compared to ipilimumab alone. This combination therapy demonstrated enhanced antitumor activity without introducing any additional safety concerns (199). Meng Y (200) constructed a novel dual-gene recombinant oncolytic adenovirus called RCAd-LTH-shPD-L1 based on the RCAd virus platform, emphasizing the potential of combining local virotherapy and antiangiogenic therapy with ICIs as an effective TME therapy for the treatment of poorly infiltrating tumors. Additionally, the MASTERKEY-265 trial, a Phase Ib/III investigation, assessed the effectiveness of T-VEC in combination with pembrolizumab for individuals with unresectable stage IIIB-IVM1c melanoma. The study demonstrated enhanced antitumor activity and verified that there were no unidentified safety concerns (201).

### 4.5 The development of individualized treatment strategies

The personalized approach to tumor treatment, often known as precision medicine, entails developing a customized therapeutic plan for every patient, grounded in their distinctive biological traits, genomic data, tumor features, and the associated microenvironment. This method seeks to improve the effectiveness of treatment while reducing avoidable side effects by more precisely pinpointing the particular tumor type and its possible weaknesses.

#### 4.5.1 Antiangiogenic drugs selected according to the molecular characteristics of the tumor

The initial strategy emphasizes vascular endothelial growth factor (VEGF) along with its receptors. One can evaluate the expression levels of VEGF or its receptors through immunohistochemistry (IHC) (202, 203) or molecular biology assays (204). In tumors, heightened levels of VEGF or its receptors often indicate a likelihood of responding to anti-VEGF therapies (205). The second approach examines the characteristics of the tumor microenvironment. Elevated levels of inflammatory markers, such as T cells, in certain tumors may suggest a greater responsiveness to anti-angiogenic drugs (206). The third approach is based on molecular markers. Mutations in specific oncogenes (e.g., HRAS, KRAS) (207) may be associated with sensitivity to anti-angiogenic therapies, necessitating the monitoring of these genetic statuses to guide drug selection. Additionally, some cancer cells harbor mutations in their signaling pathways (such as PI3K/AKT/mTOR) (208), indicating the potential need for combination therapies involving anti-angiogenic drugs and agents targeting these pathways. Lastly, the development of targeted therapies focuses on tumor-specific molecular features, such as particular gene mutations (209). These targeted agents can selectively affect tumor cells while minimizing impact on normal cells, exemplified by drugs like EGFR inhibitors and ALK inhibitors.

#### 4.5.2 Development of treatment plan based on patient genetic background

Initially, tumor genome sequencing (210) can be conducted. Through comprehensive genomic sequencing of patient tumor tissue, we can identify mutations, copy number variants, gene rearrangements, and other genomic alterations (211). These data are instrumental in defining the molecular characteristics of tumors, identifying driver mutations, and pinpointing potential drug targets (212, 213). By pairing a driver mutation with an appropriate drug, we can tailor treatments for molecularly complex and heterogeneous cancers using a combination of customized therapies (214). Song et al. (215) demonstrated for the first time that treatment plans for bispecific antibodies can be customized for advanced non-small cell lung cancer based on the genetic information of the stromal region, with the potential to predict treatment efficacy.

Additionally, we can perform a liquid biopsy, which provides non-invasive options for tumor detection and personalized therapy by detecting circulating tumor DNA (ctDNA) and other biomarkers in cells. Some studies have indicated that urine DNA methylation detection can facilitate early detection and recurrence monitoring of bladder cancer (216).

Moreover, we can develop individualized vaccines based on tumor-specific antigens to activate and enhance the patient’s immune response. The high tumor specificity and immunogenicity of these vaccines are considered the ultimate goal of tumor immunotherapy. New antigen-based dendritic cell (DC) vaccines represent a promising therapeutic modality. One investigator initiated a single-arm pilot study (ChiCTR-O NC-16009100, NCT02956551) using a personalized neoantigen peptide-pulsed autologous DC vaccine for lung cancer.

Furthermore, screening for biomarkers is crucial. Patients most likely to benefit from treatment can be selected based on their tumor biomarkers, such as PD-L1 expression, DNA repair defects (e.g., BRCA mutations), and fusion gene status. Lastly, a risk assessment can be performed by evaluating genetic susceptibility, family history, and other factors to customize surveillance and prevention strategies (217, 218).

## 5 Conclusion and outlook

Anti-angiogenic drugs have demonstrated remarkable efficacy in the clinical treatment of cancer; however, they continue to encounter numerous limitations and challenges, including response rates, drug resistance, toxicity, side effects, and individual patient variability. A primary concern is the low response rate associated with these therapies. While the efficacy of certain anti-angiogenic agents, such as bevacizumab and sorafenib, is notable in specific cancer types, the overall response rate remains relatively low, resulting in many patients failing to derive significant benefits. Anti-angiogenic agents work by inhibiting tumor growth and dissemination through the disruption of blood supply. Nonetheless, prolonged use can lead to adverse long-term outcomes, including an increased risk of hypoxia-induced local tumor invasion, distant metastasis, revascularization, and tumor recurrence after discontinuation of therapy (219). Additionally, immune evasion by tumor cells significantly contributes to the reduced effectiveness of anti-angiogenic drugs. For instance, Zhang et al. (220) found that inhibition of ALDH2 resulted in decreased PD-L1 protein levels in colorectal cancer (CRC) cells, thereby promoting the infiltration of tumor-infiltrating lymphocytes (TILs) and facilitating the escape of colorectal tumors from immune surveillance. Furthermore, the mechanisms underlying drug resistance are multifaceted. Resistance to anti-angiogenic therapies can be either innate, linked to the host, or acquired by tumor cells. Following anti-angiogenic treatment, tumors may develop resistance through various mechanisms, ultimately leading to treatment failure (221, 222). Examples include the activation of alternative angiogenic pathways, alterations in tumor cell surface antigens, and enhancement of the tumor microenvironment (223–225). Moreover, elements like genetic alterations, vascular mimicry, co-selection of vascular components, and intussusception angiogenesis could also play a role in the emergence of drug resistance (219). Finally, the side effects. Antiangiogenic drugs in the inhibition of tumor angiogenesis, may affect the normal physiological blood vessels, hypertension (226), skin adverse reactions (such as alopecia, hairy, papules pus rash, etc.) (227), cardiotoxicity (228), bleeding, wound healing (229), and jaw bone necrosis (ONJ) (230). Rare adverse reactions such as gastrointestinal tract perforation (231). In addition, the different physical conditions, complications and tumor characteristics of different patients cause great changes in the efficacy and tolerance of drugs (232).

Future research on antiangiogenic drugs can be categorized into several key areas. First, there is a need for the development of new drugs and the identification of novel targets. This includes the creation of more selective and specific anti-angiogenic agents, cancer vaccines, and the exploration of drugs that can simultaneously act on multiple signaling pathways. Second, combination therapy should be emphasized. Antiangiogenic drugs can be effectively combined with modalities such as cell therapy or chemotherapy to enhance overall efficacy. Individualized treatment approaches are also being pursued. With the advancement of biomarkers, genomic analysis, and liquid biopsy methods, as well as molecular monitoring technologies, it becomes possible to track alterations in tumor genomics and evaluate treatment impacts and necessary modifications in a dynamic manner. Ultimately, innovation in technology is essential for future progress. This encompasses the creation of sophisticated targeted drug delivery systems to guarantee that antiangiogenic medications effectively arrive at tumor locations, thereby reducing systemic adverse effects. At the same time, it is important to develop biological imaging technologies that can monitor the distribution and effectiveness of drugs within the body in real-time, facilitating the assessment of treatment responses and the fine-tuning of treatment strategies.

Considerable opportunities exist for the future progression of antiangiogenic medications. Continuous improvements in technology, new drugs, and creative therapeutic approaches are expected to boost the effectiveness and safety of antiangiogenic treatments. With extensive biological studies and thorough clinical trials, it is anticipated that patients will receive more accurate, convenient, and efficient treatment alternatives.
